# Supplementary material for: Balloon-expandable versus self-expanding transcatheter aortic valve replacement for bioprosthetic dysfunction: A systematic review and meta-analysis
Source: PLoS One. 2020 Jun 1;15(6):e0233894. doi: 10.1371/journal.pone.0233894 (PMC7263630; doi:10.1371/journal.pone.0233894)
Supplement: S1 Appendix — (DOCX) [file pone.0233894.s001.docx]

**S2 Appendix**: detailed search strategy

1. (“valve in valve”[Title] AND “aortic”[Title])
2. (“transcatheter” [Title] AND “aortic” [Title] AND “valve” [Title])
3. (“failed” [Title] or “failing” [Title] or “degenerated” [Title] or “degenerative” [Title] or “degeneration” [Title] or “deterioration” [Title])
4. (2) AND (3)
5. (1) OR (4)
6. Limit (5) to “English language” and “journal article”

Embase

1. **'valve in valve'**:ti AND **aortic**:ti
2. **transcatheter**:ti AND **aortic**:ti AND **valve**:ti AND (**failed**:ti OR **failing**:ti OR **degenerated**:ti OR **degenerative**:ti OR **degeneration**:ti OR **deterioration**:ti)
3. (#1 OR #2) AND ([article]/lim OR [article in press]/lim) AND [english]/lim

Ovid

1. ("valve in valve" and aortic).m_titl.
2. (transcatheter and aortic and valve and (failed or failing or degenerated or degenerative or degeneration or deterioration)).m_titl.
3. 1 or 2
4. limit 3 to (english language and humans and journal article)

Pubmed
(("valve in valve"[Title]) AND (aortic[Title]) AND ((journalarticle[Filter]) AND (english[Filter]))) OR ((((transcatheter[Title]) AND (aortic[Title])) AND (valve[Title]) AND ((journalarticle[Filter]) AND (english[Filter]))) AND (“failed” [Title] or “failing” [Title] or “degenerated” [Title] or “degenerative” [Title] or “degeneration” [Title] or “deterioration” [Title] AND ((journalarticle[Filter]) AND (english[Filter]))) AND ((journalarticle[Filter]) AND (english[Filter])))
