# Supplementary material for: Balloon-expandable versus self-expanding transcatheter aortic valve replacement for bioprosthetic dysfunction: A systematic review and meta-analysis
Source: PLoS One. 2020 Jun 1;15(6):e0233894. doi: 10.1371/journal.pone.0233894 (PMC7263630; doi:10.1371/journal.pone.0233894)
Supplement: S2 Table — (DOCX) [file pone.0233894.s003.docx]

**Supplementary Table.** Newcastle-Ottawa Scale Quality Assessment of included studies

|  |  | | | |  | | | | |
| --- | --- | --- | --- | --- | --- | --- | --- | --- | --- |
|  | **Selection** | | | | **Comparability** | **Outcome** | | |  |
| **First author / Year** | **Representativeness of the exposed cohort** | **Selection of the non-exposed cohort** | **Ascertainment of exposure** | **Outcome of interest not present at start of study** | **Comparability** | **Assessment of outcome** | **Was follow-up long enough for outcomes to occur?** | **Adequacy of follow -up of cohorts** | **Total** |
| Woitek/2020 | ★ | ★ | ★ | ★ | ★ | ★ | ★ | ★ | 8 |
| Ribeiro/2018 | ★ | ★ | ★ | ★ | ★ | ★ | ★ | ★ | 8 |
| Ochiai/2018 | ★ | ★ | ★ | ★ | ★★ | ★ | ★ | ★ | 9 |
| Dvir/2014 | ★ | ★ | ★ | ★ | ★ | ★ | ★ | ★ | 8 |
| Ihiberg/2013 | ★ | ★ | ★ | ★ | ★ | ★ | ★ | ★ | 8 |
| Stankowski/2020 | ★ |  | ★ | ★ |  | ★ | ★ | ★ | 6 |
| Pascual/2019 |  |  | ★ | ★ |  | ★ | ★ | ★ | 5 |
| Schwerg/2018 | ★ |  | ★ | ★ |  | ★ | ★ | ★ | 6 |
| Scholtz/2018 | ★ |  | ★ | ★ |  | ★ | ★ | ★ | 6 |
| Sang/2018 |  |  | ★ | ★ |  | ★ | ★ | ★ | 5 |
| Deeb/2017 | ★ |  | ★ | ★ |  | ★ | ★ | ★ | 6 |
| Chhatriwalla/2017 |  |  | ★ | ★ |  | ★ | ★ | ★ | 5 |
| Duncan/2015 | ★ |  | ★ | ★ |  | ★ | ★ | ★ | 6 |
| Ong/2012 | ★ |  | ★ | ★ |  | ★ | ★ | ★ | 6 |
| Linke/2012 | ★ |  | ★ | ★ |  | ★ | ★ | ★ | 5 |
| Bedogni/2011 | ★ |  | ★ | ★ |  | ★ | ★ | ★ | 6 |
| Murdoch/2020 |  |  | ★ | ★ |  | ★ | ★ | ★ | 5 |
| Stankowski/2019 |  |  | ★ | ★ |  | ★ | ★ | ★ | 5 |
| Seiffert/2018 | ★ |  | ★ | ★ |  | ★ | ★ | ★ | 6 |
| Webb/2017 | ★ |  | ★ | ★ |  | ★ | ★ | ★ | 6 |
| Nielsen-Kudsk/2017 |  |  | ★ | ★ |  | ★ | ★ | ★ | 5 |
| Ye/2015 | ★ |  | ★ | ★ |  | ★ | ★ | ★ | 6 |
| Bapat/2014 | ★ |  | ★ | ★ |  | ★ | ★ | ★ | 6 |
| Seiffert/2012 | ★ |  | ★ | ★ |  | ★ | ★ | ★ | 6 |
| Bapat/2012 | ★ |  | ★ | ★ |  | ★ | ★ | ★ | 6 |
| Pasic/2011 | ★ |  | ★ | ★ |  | ★ | ★ | ★ | 6 |
| Kempfert/2010 | ★ |  | ★ | ★ |  | ★ | ★ | ★ | 6 |
